# Biomolecule-Mediated Therapeutics of the Dentin–Pulp Complex: A Systematic Review

**DOI:** 10.3390/biom12020285

**Published:** 2022-02-09

**Authors:** Foteini Machla, Ioannis Angelopoulos, Matthias Epple, Maria Chatzinikolaidou, Athina Bakopoulou

**Affiliations:** 1 Department of Prosthodontics, School of Dentistry, Faculty of Health Sciences, Aristotle University of Thessaloniki (A.U.Th), 54124 Thessaloniki, Greece; fcmachla@dent.auth.gr; 2 School of Dentistry, Faculty of Health Sciences, Aristotle University of Thessaloniki (A.U.Th), 54124 Thessaloniki, Greece; iangelopoulos84@gmail.com; 3 Inorganic Chemistry and Center for Nanointegration Duisburg-Essen (CeNIDE), University of Duisburg-Essen, 45117 Essen, Germany; matthias.epple@uni-due.de; 4 Department of Materials Science and Technology, University of Crete, 70013 Heraklion, Greece; mchatzin@materials.uoc.gr; 5 Institute of Electronic Structure and Laser, Foundation for Research and Technology-Hellas, 70013 Heraklion, Greece

**Keywords:** dentin-pulp complex regeneration, signaling molecules, cell homing, tissue engineering

## Abstract

The aim of this systematic review was to evaluate the application of potential therapeutic signaling molecules on complete dentin-pulp complex and pulp tissue regeneration in orthotopic and ectopic animal studies. A search strategy was performed according to the Preferred Reporting Items for Systematic Reviews and Meta-Analyses (PRISMA) statement in the MEDLINE/PubMed database. Animal studies evaluating the application of signaling molecules to pulpectomized teeth for pulp tissue or dentin-pulp complex regeneration were included. From 2530 identified records, 18 fulfilled the eligibility criteria and were subjected to detailed qualitative analysis. Among the applied molecules, basic fibroblast growth factor, vascular endothelial growth factor, bone morphogenetic factor-7, nerve growth factor, and platelet-derived growth factor were the most frequently studied. The clinical, radiographical and histological outcome measures included healing of periapical lesions, root development, and apical closure, cellular recolonization of the pulp space, ingrowth of pulp-like connective tissue (vascularization and innervation), mineralized dentin-like tissue formation along the internal dentin walls, and odontoblast-like cells in contact with the internal dentin walls. The results indicate that signaling molecules play an important role in dentin/pulp regeneration. However, further studies are needed to determine a more specific subset combination of molecules to achieve greater efficiency towards the desired tissue engineering applications.

## 1. Introduction

According to the General Assembly of the United Nations in September 2011, oral diseases are recognized as a major health burden for many countries. During this meeting, a consensus was reached to include oral diseases in the common response to Non-Communicable Diseases (NCDs), as they share several common risk factors [1]. Dental diseases, as part of the oral disease family, are not life-threatening and are mostly preventable. However, their therapeutic management is a global health priority issue due to their very high prevalence. More specifically, 3.5 billion cases (45% of the general population) suffer from various oral conditions, such as caries, periodontal disease, edentulism, or other oral diseases (e.g., oral cancer, myalgia, genetic diseases). Moreover, 2.3 billion cases (29%) have untreated caries in permanent teeth [2]. Considering the constant aging of the population in high/middle income countries, the prevalence of dental diseases is expected to increase even more. It has also been found that oral conditions, especially dental caries, negatively impact the Health-Related Quality of Life in adults (HRQoL) [3]. It is estimated that the worldwide costs attributed to dental diseases were USD 544.41 billion in 2015, of which 12% were due to untreated dental caries [4].

Nowadays, conservative dentistry primarily uses various biomaterials to replace the diseased/defected dental tissues. Nevertheless, replacing a tissue by inert biomaterials is associated with many disadvantages, such as deterioration of the material over time in terms of its aesthetical and mechanical properties, loss of functionality, and loss of sensation of the restored tooth [5]. Therefore, research interest has recently targeted regenerative and tissue engineering-based treatment approaches.

The structure of the tooth is unique compared to other parts of the human body. Externally, teeth consist of hard tissues, i.e., enamel that covers the tooth crown, cementum that covers the root, and dentin that lies underneath these two. Dentin surrounds the internal cavity of the tooth, which contains the soft pulp tissue [6]. Dentin is a bone-like tissue occupying the biggest part of the tooth volume, and has a specific microstructure, where dentinal tubules connect the pulp to the outer shell [6]. Pulp is a connective tissue consisting of nerves and vessels connected to the circulatory system by the apical foramen. The outer pulp zone constitutes a monolayer of odontoblasts, specialized cells for the sensation of the teeth. Odontoblasts consist of a cell body that is attached to the inner dentin wall and their processes run through the dentinal tubules [7]. Brannstrom’s hydrodynamic theory suggests that dentinal and pulpal nerve endings are stimulated by the rapid movement of the liquid inside the dentinal tubules caused by thermal, chemical, and microbial stimuli [8]. The spatial restrain of the pulp plays a significant role in dental pathoses, because even when a mild damaging stimulus reaches the pulp, it causes inflammation, and the inability of the pulp’s expansion often leads to its necrosis. In general, dental tissues do not possess regenerative properties, aside from the limited ability of the dentin-pulp complex, which results in the development of secondary dentin formation externally of the pulp.

In the past few decades, many innovations have been reported regarding the regeneration of various tissues and organs including oral and dental tissues. Tissue engineering (TE) is a promising innovative approach that utilizes the triad of stem cells (SCs), scaffolds, and stimulating factors, aiming at the regeneration of diseased, damaged, or lost tissues and organs [9]. Various dental tissue-derived mesenchymal stem cells (MSCs) have been discovered and studied, e.g., dental pulp SCs from permanent (DPSCs) teeth or deciduous (SHED) teeth, SCs from the apical papilla (SCAP), periodontal ligament SCs (PDLSCs), and orofacial bone marrow MSCs (BMMSCs) [10]. In addition to this, dental SCs are being investigated for the regeneration of non-dental tissues [11]. Stem cells can be transplanted to the desired area (cell-based approach) or the already-existing endogenous host’s SCs can be activated through signaling, recruited, and differentiated into relevant cell types (so-called cell-homing approach). Dental research has only recently navigated towards cell-free approaches, mainly to avoid the ethical and legal limitations of the cell-based option that arise from the potential risk of immunogenic reaction and tumorigenesis [12]. A large variety of scaffolds have already been evaluated in dental tissue engineering. These scaffolds should be biocompatible, biodegradable, and promote cell proliferation and differentiation. Synthetic, natural, and smart biomaterials have been manufactured and tested, revealing promising results [13].

Stem cell differentiation is affected by intrinsic stimuli in which the transcription factors are expressed by cells, and by extrinsic stimuli with the mechanical and chemical signals provided by the extracellular matrix, growth factors, and neighboring cells [14]. The study of the stages of odontogenesis has revealed the major role of signaling molecules on stem cell fate. Many signaling molecules have been recorded and their role in dental regeneration is partially clarified [15]. Among others, growth factors (GF) such as fibroblast GF–2 (FGF2), platelet-derived GF (PDGF), and vascular endothelial GF (VEGF), are related to angiogenesis; nerve GF (NGF) to neuronal growth; transforming GF-b1 (TGFb1), stromal cell-derived factor-1 (SDF1), FGF2, and PDGF to chemotaxis; Bone Morphogenetic Protein-2, -4, -7, and -11 (BMP-2, -4, -7, -11), insulin-like GF (IGF), TGFb1, PDGF, FGF2, and NGF to differentiation; and PDGF, FGF2, IGF, VEGF, TGFb1, and SDF1 to proliferation [16]. Most of the above-mentioned signaling molecules are fossilized in dentin tissue. Therefore, suggestions on using chelating agents, e.g., EDTA, to release these molecules from the dentin tissue have been described in the literature, leading to a successful release and treatment outcome [17].

This systematic review based on animal studies elucidates the efficacy of signaling molecules in combination with or without scaffolds, on the cell-free regeneration of the pulp tissue or of the entire dentin-pulp complex.

## 2. Materials and Methods

This systematic review was reported following the Preferred Reporting Items for Systematic Reviews and Meta-Analyses (PRISMA) statement [18]. Based on the PICO principle (Population, Intervention, Comparison, and Outcome), the following review question was formulated: Does the therapeutic application of biomolecules assist dentin/pulp regeneration through cell homing?

### 2.1. Search Strategy

Two independent reviewers performed the literature search in the MEDLINE database through PubMed search engine. In case of disagreement, a consensus between the two reviewers was reached after thorough analysis of the data. The search was performed until November 2021, without any other limit set for the publication date. The search strategy is presented in Table 1.

### 2.2. Study Selection

Studies that were stated as retracted, and reviews or studies that were written in any other language than English, were not screened. Two authors screened the titles and abstracts of the retrieved articles independently and the full texts of the potentially eligible studies were assessed. The eligibility criteria addressed original papers of animal studies in the English language that evaluated signaling molecules in the dentin–pulp complex or pulp tissue complete regeneration via cell homing. The identification of the included articles was not limited by the presence or absence of scaffolds/carriers/delivery systems, the type of signaling molecules, or the application in ectopic/orthotopic animal models. In vitro studies were excluded from this systematic review. In vivo studies that evaluated dentin/pulp regeneration through cell-based (i.e., cell transplantation) techniques were excluded, even if biomolecules were applied. Studies on partial regeneration (pulp capping or partial pulpotomy) were excluded. Review articles, editorial letters, short communications, case reports, and case series were also excluded.

### 2.3. Data Extraction

The following data were retrieved from all included studies: author; study model (orthotopic, ectopic); animal and replicate number; tooth information; biomolecules applied and their concentration; restorative material; treatment duration; evaluation methodology; and main results.

### 2.4. Risk of Bias

The evaluation of risk of bias of the included studies was based on the SYRCLE’s tool for animal studies, developed by the Systematic Review Centre for Laboratory Animal Experimentation [19]. The following parameters were considered: random sequence generation (selection bias); baseline characteristics (selection bias); blinding of outcome assessment (detection bias); incomplete outcome data (attrition bias); selective reporting (reporting bias); and other bias (design-specific risks). Each component was classified as low, unclear, or high risk of bias.

## 3. Results

### 3.1. Study Selection

**Figure 1:**
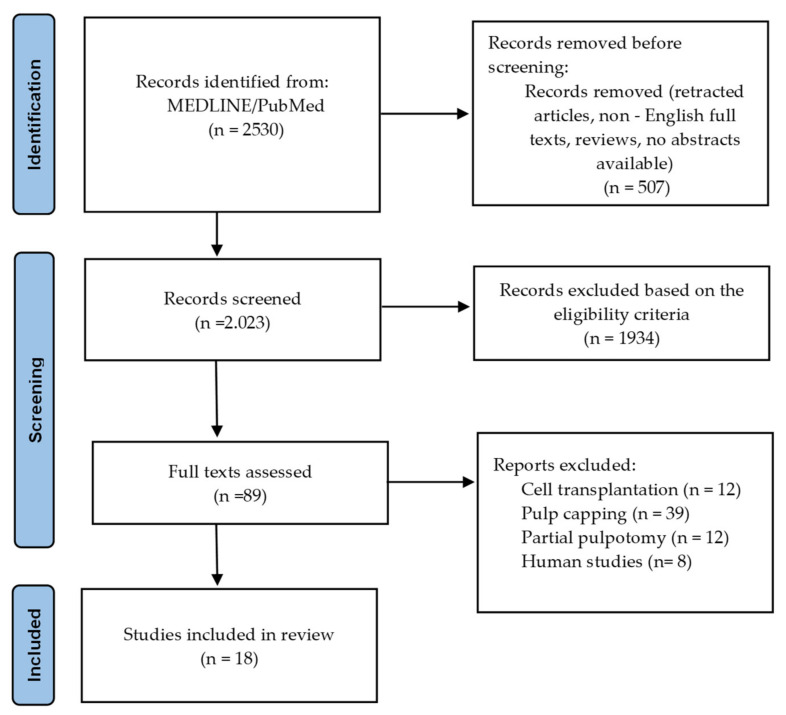
PRISMA flow diagram.

The PRISMA flow diagram depicting the path of information through the different phases of the systematic review is shown in Figure 1. A total of 2530 records were identified, 507 of which were not screened (retracted articles, reviews, non-English texts, non-available abstracts). Out of the 2023 potentially relevant articles that were screened, 1934 were excluded based on the eligibility criteria. Full texts of 89 studies were assessed, 71 of which were excluded due to the following exclusion criteria: partial pulp regeneration studies (pulp capping and partial pulpotomy) (n = 51); cell transplantation studies (n = 12); and studies in humans (n = 8).

### 3.2. Descriptive Analysis

Out of the 18 included studies on complete dentin–pulp complex or pulp tissue regeneration, 10 used an orthotopic model (teeth of the animal were treated), while 8 used an ectopic model (allogenic teeth were extracted, treated, and transplanted subcutaneously in another animal). The information collected from the orthotopic and ectopic studies included in this systematic review can be seen in Table 2 and Table 3, respectively. The most frequently used animal type in orthotopic studies was dogs (70%), while ferrets, minipigs and rats were used in the rest of the studies. In the ectopic model, 75% of the studies were performed on mice and the rest on rats. The transplanted teeth originated from humans (87.5%), with only one study involving porcine teeth. The age, sex, and weight of the animals were the most frequently stated animal characteristics.

In 5 out of 10 orthotopic studies, pulpitis/pulp necrosis was induced before the treatment. Sodium hypochlorite (NaOCl), at a range of concentrations, was chosen in 9 out of 10 orthotopic (from 0.5 to 5.25%) and in 4 out of 8 ectopic studies (1.5 to 5.25%) to disinfect the root canals. Teeth were autoclaved in 3 out of 8 ectopic studies. For further disinfection, smear layer removal and demineralization of the intracanal dentin, EDTA 17% was used in 6 orthotopic and 4 ectopic studies. EDTA was either washed off or used as the final step of the chemo-mechanical preparation of the roots. In 5 out of 10 orthotopic studies, mature (closed apex) teeth were used, while in the rest immature (open apex) teeth were used. In all orthotopic cases, where an induced pulp infection was established, triple antibiotic paste (TAP) was applied in the root canals between the sessions, until the final stage of regenerative endodontic procedure was performed. In order to accommodate an easier recruitment of the extracanal cells into the root canal, the dental apical foramen was widened by mechanical means, e.g., K files in 40% of the orthotopic and in 25% of the ectopic studies. In ectopic studies, mostly decoronized teeth/roots or dentin cylinders were transplanted, with sealed or non-sealed coronal opening. The most common (60%) sealing material of the orthotopic studies was mineral trioxide aggregate (MTA), followed by glass ionomer cement (GIC) (20%) and composite resin (20%). The duration of orthotopic treatment ranged from 1 to 4 months, while ectopic studies lasted from 3 to 12 weeks. The endpoints of the treatment were evaluated in all studies by histology, followed by immunohistochemistry (IHC), radiographs, and in few by Polymerase Chain Reaction (PCR) analysis.

Regarding the signaling molecules that were applied for the complete dentin/pulp regeneration, bFGF and VEGF were each applied in 5 studies, BMP7 and Platelet-Rich Plasma (PRP) in 3 studies, and PDGF and NGF in 2 studies. TGF-b1, Dentin Matrix Protein–1 (DMP1), Concentrated GF (CGF), SDF-1a, Stem Cell Factor (SCF), dental pulp extracellular matrix (DP-ECM), chitosan, self-assembling peptides (SAPs), Wnt signaling protein 3a (Wnt3a), and Granulocyte-Colony Stimulating Factor (G-CSF) were each used in 1 study. One orthotopic and four ectopic studies included stem cell transplantation; these studies compared the cell-free approach to a cell-based model. Therefore, these studies were included since the cell transplantation was not evaluated as the main tissue engineering factor.

The clinical, radiographical and histological outcome measures of the studies included: healing of periapical lesions (when pulp infection was previously induced); root development and apical closure (when immature teeth were evaluated); cellular recolonization of the pulp space; ingrowth of pulp-like connective tissue; vascularization and innervation in the pulp space; mineralized dentin-like tissue formation along the internal dentin walls; and odontoblast-like cells in contact with the internal dentin walls. More information on the results of the included studies can be seen in Table 2 and Table 3. In some cases, bone- and cementum-like tissue, as well as fibrodentin, were observed.

### 3.3. Risk of Bias Assessment

**Figure 2:**
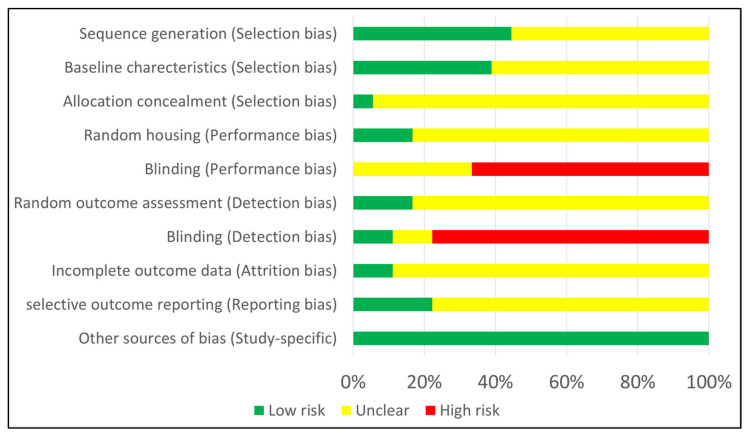
Risk of bias using SYRCLE’s tool for animal studies.

The risk of bias assessment of the included studies is depicted in Figure 2. The studies scored moderately on the selection bias component, where most of the studies stated ‘samples/animals were randomly divided into groups’, without providing information about the randomization process. Only one study provided information about controlled housing and two about same housing conditions among groups. Performance and detection blinding was rarely stated, while only a few studies reported the number of samples that were eventually assessed along with the missing values. No design-specific risk was observed among the studies.

## 4. Discussion

Conservative dentistry treats irreversible pulpitis and pulp necrosis by replacing the defected pulp tissue with root canal-filling materials e.g., gutta-percha. Pulp replacement by inert biomaterials is bound to multiple limitations, the most prevalent being tooth sensation loss and tooth brittleness, which results in vulnerability to fractures. The goal of TE approaches is for defected tissues to be regenerated and not to be replaced permanently by inert biomaterials. To this end, stem cells, scaffolds, and stimulating factors, including signaling molecules, are proposed in the context of a regenerative approach [38].

The initial discovery of DPSCs by Gronthos et al. in 2000, along with the later discovery of SHEDs and SCAPs, led to an increased research interest around dentin/pulp tissue regeneration, with many in vitro studies already published [39,40,41]. Due to their origin, these cells have the ability to differentiate into odontoblasts and neural and endothelial cells [42]. The two main approaches of dentin–pulp complex regeneration include cell-based and cell-free techniques. In cell-based techniques, exogenous, autologous, or allogeneic stem cells are collected, processed in vitro, and later transplanted into the area of interest [43]. In cell-free approaches, endogenous stem cells—that often reside in perivascular niches—are chemotactically activated, recruited to the area of interest, and differentiated into the relevant cell types [44]. Even though many cell-based studies in animals, and few recently published in humans, have shown promising results in dentin-pulp regeneration, the potential risks and handling as well as the cost-related limitations of stem cell therapies, have led to a proposed shift towards the cell-free approach [45,46,47].

Dentin and pulp regeneration are complex and highly organized by signaling molecules. Our study evaluated the advantageous effect of additionally applied signaling molecules to dentin-pulp complete regeneration in animal studies. Fully pulpectomized teeth, roots, and dentin cylinders in orthotopic or ectopic animal models were evaluated; studies on partial regeneration (pulp capping or partial pulpotomy) were excluded.

Several signaling molecules have been proposed to support this concept, including FGF2, PDGF, VEGF, NGF, TGFb1, SDF1, BMP2/4/7/11, and IGF. Among those, Fibroblast growth factors (FGF) form a family of 22 polypeptides, of which bFGF is the most frequently investigated in dentin/pulp regeneration. BFGF is involved in all stages of pulp repair/regeneration and uses multiple signaling pathways to control the relevant biological procedures [48]. BFGF binds to tyrosine kinase FGF receptors 1–4 on the cell membrane, and phosphorylation occurs. Then, a cascade of intracellular signaling pathways, such as the RAS-MAPK, PI3K-AKT, PLCγ, and JAK/STAT, initiate cell migration, proliferation, and differentiation [49]. Five included studies used bFGF alone or in combination with other signaling molecules for pulp regeneration [22,31,32,34,35]. Suzuki et al. transplanted ectopically human teeth treated with bFGF in collagen gel subcutaneously in the dorsum of rats for 3 weeks. Histological assessment revealed red pigmentation, recellularization, and tissue integration compared to the negative control group [35]. Takeuchi et al. transplanted porcine roots, treated with bFGF or G-CSF in collagen, subcutaneously in mice for 21 days, resulting in similar regeneration of pulp-like tissue, intracanal cell density, and newly formed capillaries [32]. G-CSF is a chemotactic molecule already approved by the Food and Drug Administration (FDA) for clinical use. G-CSF has been found to mobilize the migration of DPSCs of high stemness, the proliferation, and the differentiation of endogenous stem cells; to enhance vascularization and innervation; and to present anti-inflammatory and anti-apoptotic properties [50].

VEGF is characterized as an important factor with pro-angiogenic activity having a mitogenic and anti-apoptotic effect on endothelial cells and enhancing cell migration and high vascular permeability [51]. VEGFs form a family of several growth factors, namely VEGF-A, -B, -C, -D, -E, -F, -PIGF, and IG-VEGF. These factors bind to tyrosine kinase cell receptors, VEGFRs like VEGFR-1, VEGFR-2, and VEGFR-3. VEGF stimulates vascularization by activating multiple signaling pathways. VEGF-A binding on VEGF-R2 on plasma membrane of endothelial cells leads to downstream signaling of the PLCγ/PKC causing cell proliferation; of PI3K enhancing cell survival and vasodilatation permeability; and of MAPK causing cell migration [51]. Five included studies investigated the role of VEGF (VEGF-A and -C) alone or in combination with other signaling molecules in dentin/pulp regeneration [22,30,31,33,34]. Li et al. transplanted full-length human roots treated with VEGF-loaded heparin-conjugated gelatin nanospheres immobilized in PLLA microspheres (HG-MS) subcutaneously in mice for 9 weeks. The results showed minimal soft tissue formation, but to a lesser extent compared to DPSCs transplantation along with VEGF [30]. Yadlapati et al. transplanted human roots treated with VEGF-A in polydioxanone fibers subcutaneously in mice, resulting in new blood vessels and connective tissue formation at 45 days [33].

BMPs constitute a family of proteins with a major role in tooth development, initiation, morphogenesis, cytodifferentiation, and matrix secretion acting through complex signaling networks [52]. BMP signaling pathway is regulated by intracellular domains, membrane sites or extracellular sites. Through two transmembrane receptors, type I and type II, with serine-threonine kinase activity expressed in dental pulp, BMP signals are transferred to the nucleus by Smad proteins, receptor-activated Smads (R-Smads), common mediator Smads (co-Smads), and inhibitory Smads (I-Smads). Three included studies evaluated the role of BMP7 alone or in combination with other signaling molecules [22,28,31]. He et al. treated minipig teeth with BMP7 in collagen gel for 3 months, resulting in excessive mineralization with embedded cells resembling bone. Though, when BMP7 was combined with Wnt3a application, vascularized dental pulp-like tissue and mineralized dentin-like tissue with dentinal tubules and neural filament-like structures were observed [28]. The Wnt family, consisting of 19 cysteine-rich glycoprotein members, is important in regulating cellular functions, self-renewal, proliferation, differentiation, and motility. Wnt signaling is a fundamental signaling in dentin/pulp regeneration controlling cell proliferation, differentiation, polarization, and apoptosis. Wnt3a acts through the canonical Wnt signaling pathway where Wnt3a binds to its receptor Frizzled (Fz) and co-receptor lipoprotein receptor-related protein (LRP5/6), and then Disheveled (Dvl) protein is activated. The complex of casein kinase 1 (CK1), glycogen synthase kinase-3β(GSK-3β) and the adenomatous polyposis coli (APC) proteins, Axin1 and Axin2, are dissociated leading to β-catenin degradation. The transportation of β-catenin into the nucleus regulates the expression of Wnt genes [53].

NGF is the most frequently investigated neurotrophic factor that promotes neuritis outgrowth in vivo and in vitro [54]. NGF binds to the specific receptor tropomyosin kinase receptor A (TrkA) activating the signaling pathways of MAPK, ERK, PI3K, PLC, and to the non-selective P75 pan-neurotrophin receptor (p75^NTR^) activating Jun kinase signaling cascade, NF-κΒ and ceramide generation [55]. PDGF signaling contains four ligands, PDGFA-D and two tyrosine kinase receptors, PDGFR-α, and -β. PDGF-BB has been found to act as a chemoattractant by activating the PI3K/Akt signaling pathway to enhance DPSC proliferation, angiogenesis, and odontogenic differentiation, while having the ability to induce cell homing and support dentin–pulp complex regeneration in vivo [56]. Two included studies evaluated the combined use of NGF and PDGF with other signaling molecules in dentin/pulp regeneration [22,31]. El Ashiry et al. treated immature human apical pulpitis-induced teeth with VEGF, bFGF, VEGF, PDGF, NGF, and BMP7 in chitosan hydrogels for four months, while Kim et al. transplanted human teeth treated with the same combination of factors subcutaneously in mice for three weeks [22,31]. In the first study, periapical lesions were healed, radicular thickening, root lengthening, and apical closure were observed, but no dentin/pulp-like tissue was formed. The second study observed recellularization along the entire root canal, blood vessel-like structure, and some ECM formation.

The following signaling molecules were used only in one included study each: CGF, TGF-β1, DMP1, and SCF [20,34,36,37]. TGF-β1 supports cell proliferation and collagen production. TGF-β1 induces odontogenic differentiation by activating the ALK5/Smad2/3, TAK1, p38, and MEK/ERK signaling pathways [57]. Galler et al. transplanted human dentin cylinders treated with TGF-β1 in combination with bFGF and VEGF in hydrogels of self-assembling multi-domain peptides (MDPs) subcutaneously in mice for 5 weeks [34]. The results indicated the formation of soft connective tissue. DMP1 is one of the dentin non-collagenous ECM proteins that has been found to regulate mineralization. DMP1 induces odontogenic differentiation and reparative dentin formation in vivo [58]. Prescott et al. transplanted human dentin cylinders treated with DMP1 in collagen scaffolds subcutaneously in mice for 6 weeks, resulting in large numbers of viable cells recruitment and collagen matrix formation in the periphery of the perforation site [36]. SCF is a chemotactic glycoprotein with the ability to recruit stem cells. It enhances cell adhesion, proliferation, migration, and angiogenesis in vitro [59]. Ruangsawasdi et al. transplanted human immature teeth treated with SCF in fibrin gels in rats for 6 and 12 weeks, displaying increased extent of tissue ingrowth, early blood vessel and immature mineral matrix formation at 6 weeks [37]. At 12 weeks, the tissue ingrowth was not significant compared to the control, but the formed tissue was more mature; hard tissue formation at the apical opening, improved revascularization and odontoblast-like processes developed into the dentinal tubules. SDF-1α is a chemotactic factor widely expressed during dental pulp inflammation. It has been shown that SDF-1/CXCR4 induces stem cell migration by activating FAK/PI3K/Akt and GSK3β/β-catenin pathways [60]. Yang et al. treated human mature apical periodontitis-induced teeth with SDF-1α in silk fibroin scaffolds subcutaneously in mice, demonstrating regenerated pulp-like connective tissue of low to moderate density with blood vessel formation, and thin layer of mineralized tissue along the intracanal dentinal walls [21].

**Figure 3:**
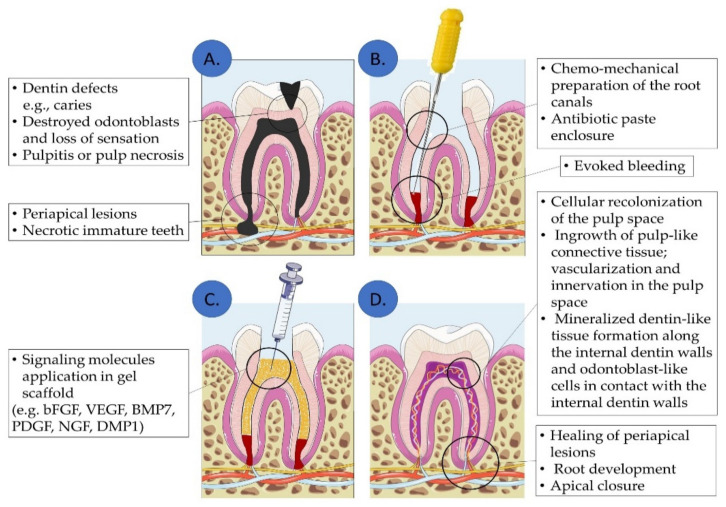
Dentin–pulp complex and pulp tissue regeneration approach by signaling molecules via cell homing: In dentin/pulp defects (**A**), the pulp chamber is chemomechanically prepared and disinfected and bleeding is evoked (**B**). Then, signaling biomolecules are applied into the root canals (**C**), leading to dentin-pulp complex and periapical tissue regeneration (**D**). (Illustration created using Servier Medical Art at smart.servier.com).

Regarding clinical practice, the most recent guidelines of the European Society of Endodontology (ESE) suggest that Regenerative Endodontic Procedures (REP) should be performed in necrotic immature teeth by evoked blood clot formation into the root canals [61]. The ultimate goal of REP would be to achieve a complete regeneration of the dentin-pulp complex and pulp tissue defects, in necrotic immature and mature teeth [17]. This systematic review provides sufficient evidence for the potential of a complete pulp regeneration with the aid of signaling biomolecules following a cell homing-based approach. (Figure 3). Initially, the root canals are chemo-mechanically prepared and disinfected. The apical foramen is widened, and bleeding is evoked, while an injectable scaffold carrying a specific subset of signaling molecules is implanted. These signaling molecules could enhance the regeneration of the target tissue. From the included studies, a variety of signaling molecules indicate the supportive role in dentin–pulp complex and pulp tissue regeneration. Further studies should be performed to specify the signaling molecules with the best potential targeted outcome and the best applicable concentration, delivery system, and duration of treatment.

## 5. Conclusions

The included orthotopic and ectopic animal studies indicate that a variety of signaling molecules have the ability to support the complete dentin–pulp complex and pulp tissue regeneration. Vascularization, innervation, intracanal dentin-like formation, or odontoblast-like cell attachment on the intracanal dentinal walls were achieved by multiple molecules and concentrations. Thus, further in vitro studies should be performed to determine a more specific subset combination of molecules for the desired outcome, as well as pre-clinical and clinical studies. Future research should focus on the clinical application of specific biomolecules, their concentrations, and their exact impact on dentin-pulp complex regeneration. The goal would be to develop a biomolecule-containing ‘cocktail’ to be applied in dentin or pulp tissues defects and to regenerate natural structure and function in dental clinical practice.

## Figures and Tables

**Table 1:** Terms for database search strategy.

PubMed Search Strategy
((dentin) OR (pulp) OR (tooth)) AND ((regeneration) OR (tissue engineering)) AND ((biomolecules) OR (growth factors) OR (signaling molecules))

**Table 2:** Orthotopic animal studies *. The studied signaling biomolecules are indicated in bold.

Author	Animal/ Sample Size	Tooth Type/ Pulp State	Chemomechanical Canal Preparation	Signaling Molecules, Groups/ Concentrations/ Restorative Material/ Duration of Treatment	Evaluation Methodology	Main Results
Xu [20]	Beagle dogs (5 months old) 3 dogs, 36 teeth (12 teeth/group)	Single-rooted immature anterior teeth Healthy pulp	1.25% NaOCl, 17% EDTA, sterile distilled water	(A) **CGF** (B) untouched teeth (C) only pulpectomized teeth 2 mm^3^ CGF fragments BP plus + GIC 8 weeks	Radiographs Histology (HE, MT) IHC (VEGF, Nestin)	Immature teeth continued to develop as in group B (ingrowth of soft tissues in the root canal, thickened internal root dentin walls, closed apex) Regenerated pulp-like tissue, vascularization, and innervation (moderate expression of VEGF and Nestin)
Yang [21]	Beagle dogs (12 months old, female) 2 dogs, 8 teeth, 16 roots (8 roots per group)	Mature, closed-apex 2nd and 3rd lower premolars Apical periodontitis induction for 90 days	(1) Widening of apical foramen up to #80 K-file, 2.5% NaOCl, 17% EDTA, sterile saline, TAP, intermediate restorative material and GIC for 4 weeks (2) 2.5% NaOCl, sterile saline, and treatment	(A) **SDF-1a** on silk fibroin scaffolds with evoked bleeding (B) only evoked bleeding (C) untouched teeth 100 ng SDF-1a in 1.5–2 mm^3^ silk fibroin scaffolds MTA + composite resin 3 months	Histology (HE, MT, or picrosirious red) IHC (CD31, PCNA, h-mit, Atg5, LC3) IF (DSP, CD31, LC3, h-mit) TEM, Protein extraction, Western blot analysis	A: primarily, regenerated connective tissue (rCT) of low to moderate density with low to moderate number of blood vessels, thin layer of raw regenerated mineralized tissue (rMT) along the dentinal walls, fibrous matrix similar to normal pulp, higher number of Atg5 or LC3 positive cells compared to N.C. B: Extensive rMT along dentinal walls and in the middle of the canal, rCT with few blood vessels Little nerve bundles in A and B groups
El Ashiry [22]	Dogs (6 months old, male) 12 dogs, 36 teeth (split-mouth design cell-based vs. cell-free)	Immature, upper permanent incisors Apical periodontitis induction for 3 weeks	(1) 2.5% NaOCl, TAP, IRM + GIC for 2 weeks (2) 2.5% NaOCl, sterile saline, and treatment	(A) **VEGF-2, bFGF, PDGF, NGF, and BMP7** in chitosan hydrogel (B) plus 2 × 10^5^ autologous DPSCs 10 ng/mL VEGF2, 100 ng/mL bFGF, 10 ng/mL PDGF, 50 mg/mL NGF, 100 ng/mL BMP7 MTA + composite resin 4 months	Radiographs (monthly) Histology (HE)	A and B: healing of radiolucency A: less radicular thickening, root lengthening, apical closure, dentin-like tissue formation, and no pulp-like tissue formation compared to Group B
Alqahtani [23]	Beagle dogs (13 months old, female) 2 dogs, 8 teeth (4–6 roots per group)	Mature molars and premolars Healthy pulp	Widening to 0.5 mm of apical foramen, 0.5% NaOCl, 17% EDTA, evoked bleeding	(A) porcine **DP-ECM** (shown to contain **Col1, DSP, DMP1, and vWF by IF, and VEGF, and TGF-b1** by ELISA) (B) collagen scaffold (C) only evoked bleeding 100 mg DP-ECM/root canal MTA + GIC 8 weeks	Histology (Goldner’s trichrome) IHC (CD31, DSP)	A: pulp-like formation, vascularization (CD31^+^ and DSP^+^ cells of canine origin) All three groups: cellular infiltration, and intracanal mineralization
Ghoddusi [24]	Mongrel dogs (6 months old) 3 dogs, 44 teeth	Immature premolars Induced pulpitis for 2 weeks	(1) 5.25% NaOCl, normal saline, TAP, temporary filling for 4 weeks (2) 5.25% NaOCl, normal saline, evoked bleeding	(A) **autologous PRP** (B) MTA control (C) parafilm PRP from 1 mL autologous blood MTA + GIC 3 months	Radiographs Histology (HE)	A and B: regenerated soft connective tissue and vessels, mineralized tissue (cementum, PDL, and bone-like tissues). No normal pulp, nerve, and dentin-like tissues observed C: no regeneration observed
Moreira [25]	Wistar rats (13 weeks old, male, 350–375 g) 34 rats, 34 teeth (split-mouth design treated vs. untreated teeth)	Upper molars Healthy pulp	1% NaOCl, 17% EDTA, widening of apical foramen	(A) **chitosan** scaffold (B) chitosan scaffold + BPMT (C) evoked bleeding + chitosan scaffold (D) evoked bleeding + chitosan scaffold + PBMT 5 μL of chitosan Temporary filling material + composite resin 4 weeks	Histology (HE) IHC (HSP-25)	A and B: poor-developed tissue formation C: avascular tissue formation D: well-developed pulp-like tissue with predentin present along the root canal walls, newly formed vessels, and HSP-25^+^ cells in contact with predentin
Siddiqui [26]	Beagle dogs (22–26 months old, male) 2 dogs, 12 teeth (split-mouth design)	Incisors Healthy pulp	5.25% NaOCl, 17%EDTA, PBS, widening of apical foramen, evoked bleeding	**Self-assembling angiogenic peptide** in hydrogel (SLan), self-assembling **dentinogenic peptide** in hydrogel (SLed), (A) SLan (B) Sled (C) SLan + SLed (1:1) (D) PBS 10–25 μL of hydrogels Composite resin 4 weeks	Histology (HE, MT) IHC (DSP, S100, PECAM)	B and D: poor disorganized tissue A and C: organized soft tissue with collagen deposition, large vessels, nerve bundles, odontoblast-like layer in the intracanal dentin, and DPS^+^ cells
Torabinejad [27]	Ferrets (70 days old, male) 6 Ferrets, 24 teeth	Immature canines Apical periodontitis induction for 3 weeks	(1) 1% NaOCl, TAP, sterile saline, temporary filling for 3 weeks (2) sterile saline, 17% EDTA, Emdogain	(A) **blood clot**/Gelfoam (B) **PRP** (C) no scaffold (D) untouched teeth Commercial products MTA + GIC 3 months	Histology (HE)	A and B: apical narrowing and hard tissue deposition similar to each other and increased compared to group C In 2/6 teeth of group A, 3/6 teeth of B, and 1/4 teeth of group C: bone- or cementum-like tissue ingrowth mixed with connective tissues in the apical third All groups: no root lengthening at any sample. Resolution of the periapical lesions in all groups except group C
He [28]	Micro-Yucatan minipigs (24 months old, 110–120 kg) 10 pigs, 58 teeth	Mandibular incisors Healthy	Sterile saline	(A) **rhWnt3a** (B) **BMP7** (C) rhWnt3a + BMP7 (all in collagen gel) (D) only collagen gel 50 ng/mL rhWnt3a, 1.25 mg/mL BMP7 Temporary filling + composite resin 3 months	Radiographs Histology (HE, MT)	A and C: vascularized dental pulp-like tissue with neural filament-like structures and mineralized dentin-like tissue with dentinal tubules and odontoblast processes in the newly formed dentin B: excessive mineralization with embedded cells resembling bone
Moradi [29]	Mixed breed dogs (6 months old, male) 2 dogs, 28 teeth	Immature premolars Induced pulp necrosis for 2 weeks	(1) Normal saline, 5.25% NaOCl, TAP for 3 weeks (2) 5.25% NaOCl, saline, evoked bleeding (BC)	(A) BC + **PRP** + MTA (B) BC + MTA (C) only induced pulp necrosis (D) untouched teeth GIC 1 month From 20 mL blood	Histology (HE) IHC (VEGF & factor VIII)	A and B: Newly formed soft connective tissue, vessels, and hard mineralized tissue formation. Microvessel density similar to each other and higher than Group D. Severe positive expressions of VEGF and factor VIII which peaked after 1 month C: no new vital tissue

* The abbreviations included in Table 2 and Table 3 are cumulatively described in the Abbreviations.

**Table 3:** Ectopic animal studies *. The studied signaling biomolecules are indicated in bold.

Author	Animal/ Sample Size	Tooth Type	Chemomechanical Canal Preparation/ Pulpitis Induction	Signaling Molecules, Groups/ Concentrations/ Restorative Material/ Duration of Treatment	Evaluation Methodology	Main Results
Li [30]	Haarlan mice (5 weeks old, subcutaneously, dorsum) 12 mice, 6 samples/group	13 mm human teeth roots	Widening to 1 mm of the apical foramen, 17% EDTA for 10′, 19% citric acid for 1′, Betadine for 30′, and 5.25% NaOCl for 15′, PBS, incubation for 3–7 days	(A) empty (B) DPSC (C) **VEGF-loaded HG-MS** (Heparin-conjugated gelatin nanospheres immobilized in PLLA microspheres) (D) VEGF-loaded HG-MS + DPSCs 100 μg rhVEGF in PBS containing 0.1% BSA at a concentration of 100 μg/mL Coronal end sealed with MTA 9 weeks	Histology (HE) IHC (h-mit, DSP, Nestin, CD31 and vWF)	A and C: minimal soft tissue formation, vascularization only at the apical third, very weak CD31, vWF, DSP, and Nestin detection B: pulp-like tissue regenerated decreased in the middle third, high expression of Nestin and DSP D: regenerated pulp-like tissue reached the coronal third of the canal, odontoblast-like cells (both cell types of human DPSC origin), many blood vessels (endothelial cells of host mouse origin) and strong vWF, CD31, DSP, and Nestin detection
Kim [31]	Haarlan mice (5–7 weeks old, male, subcutaneously, dorsum)	Human permanent maxillary and mandibular incisors and cuspids	Autoclaved	(A) **bFGF** (B) bFGF + **VEGF2** (C) **VEGF**, (D) bFGF + **NGF + BMP7** (E) VEGF2 + NGF + BMP7 (F) **PDGF** + NGF + BMP7 (all in collagen gel) (G) collagen gel without GFs 10 ng/mL VEGF2,100 ng/mL b FGF, 10 ng/mL PDGF, 50 ng/mL NGF, 100 ng/mL BMP7 in 2 mg/mL collagen gel No restorative material 3 weeks	Histology (HE) IHC (VEGF) ELISA (vWF, DSP and NGF)	A: revascularization with abundant cells and some ECM B and D: recellularization in the entire canal with blood vessel-like structures C: vascularization and connective tissue with abundant cells E: recellularization and amorphous dentin-like tissue layer on the native dentinal wall with multiple blood vessel-like structures F: recellularization along the entire canal with blood vessel-like structures and some ECM G: residual of scaffold but little cell ingrowth
Takeuchi [32]	SCID mice (5 weeks old, subcutaneously)	6 mm porcine 2nd incisor roots	Widening to 1 mm of apical foramen	(A) **bFGF** (B) **G-CSF** (C) MDPSCs (D) only collagen 15 μg/mL bFGF, 15 μg/mL G-CSF, 5 × 10^5^ MDPSC Zinc phosphate cement coronally 21 days	Histology (HE) IHC TRH-DE (enamelysin, DSPP)	A and B: Similarly regenerated pulp tissue, cell density, newly formed capillaries, enamelysin- and DSPP-positive cells, and TRH-DE but less than Group C
Yadlapati [33]	C57BL/6 mice (subcutaneously, dorsum, female) 5 mice, 10 teeth (split animal design right:left side/VF:Control)	10 mm human premolar roots	Widening to 1 mm of apical foramen, 1.5% NaOCl, 17% EDTA	**VEGF** in polydioxanone fibers (VF), (A) Human root fragments with VF (B) empty roots 12.2 ng/cm in polydioxanone fibers MTA coronally 45 days	Histology (HE)	A: new blood vessels and connective tissue in root canal at 45 days B: no new blood vessels or connective tissue
Galler [34]	Mice (8–10 weeks old, subcutaneously dorsum, female) 40 teeth	Dentin cylinders from coronal parts of human molars	5% NaOCl, 17% EDTA	**bFGF, TGF-β1, and VEGF** in hydrogel from self-assembling multi-domain peptides (MDPs), (A) MDP, No GF, No cells (B) MDP, GF, No cells (C) MDP, No GF, cells D) MDP, GF, cells 100 ng TGF-β1, 400 ng FGF2, and 100 ng/100 μL gel, 2 × 10^6^ DPSCs No restorative material 5 weeks	Histology (HE, MT) IHC (DSP, factor VIII)	A: only the MDP present and cylinder enclosed in fibrous capsule B: filled with soft connective tissue C: not enough cells to fill the cylinder and degrade the matrix, no tissue observed D: vascularized dental pulp-like soft connective tissue, collagenous ECM, cells in contact with dentin wall and cellular processes into the tubules expressing DSP, micro vessels, and endothelial factor VIII
Suzuki [35]	Rats (5–7 weeks old, subcutaneously dorsum) 4 samples	Human teeth	PBS, Autoclaved	(A) **bFGF** in collagen gel (B) only collagen gel 100 ng/mL bFGF in 2 mg/mL collagen gel No restorative material 3 weeks	Histology	A: Red pigmentation, recellularization and tissue integration compared to collagen gel group which appeared pale with residual collagen scaffold
Prescott [36]	Mice (subcutaneously, dorsum, 27 g) 20 teeth	Dentin cylinders from human 3rd molars	Autoclaved	**DMP1** in collagen scaffold, (A) MTA (B) Collagen scaffold (C) Collagen scaffold + DMP1 (D) Collagen scaffold + DPSCs + DMP1 (E) Collagen scaffold + DPSCs 1.2 mg/mL in 100 μL collagen, 5 × 10^6^ DPSCs No restorative material 6 weeks	Histology (HE, Safranin-O, von Kossa, MT)	A: few red blood cells B: degrading scaffold but not replaced by tissue, few red blood cells, and nucleated cells C: Large numbers of viable cells, some collagen matrix formation but not in the central perforation site, degrading collagen scaffold D: Degrading collagen scaffold, fibroblasts and endothelial cells, blood vessels and new collagen matrix formation E: similar to group B, some red blood and nucleated cells, degrading scaffold, no new matrix nor cells
Ruangsawasdi [37]	Sprague Dawley rats (adult, calvariae, female, 200–250 g) 20 teeth (split mouth design)	Human immature premolars	5% NaOCl, 17% EDTA	(A) **SCF** in fibrin gels (B) only fibrin hydrogel 15 μg/mL SCF GIC 6 & 12 weeks	Histology (Goldner’s trichrome) qPCR (DMP1, DSPP, Col1, NGF, and VEGF)	A: increased extent of tissue ingrowth, early blood vessel, and immature mineral matrix formation at 6 weeks compared to the control. At 12 weeks the tissue ingrowth was not significant anymore, but the tissue was more mature. Hard tissue at the apical opening, improved revascularization, and odontoblast-like processes further down the dentinal tubules. Upregulation of DMP1, Col1, and VEGF

* The abbreviations included in Table 2 and Table 3 are cumulatively described in the Abbreviations.

## Abbreviations

**Abbreviation**	**Definition**
Atg5	Autophagy related 5
APC	Autologous Platelet Concentrate
BC	Blood Clot
BMMSCs	Bone Marrow Mesenchymal Stem Cells
BMP	Bone Morphogenetic Protein
BP plus	Root canal sealer (product)
BSA	Bovine Serum Albumin
CD31	Cluster of Differentiation 31
Col1	Collagen type I
CGF	Concentrated Growth Factor
DMP1	Dentin Matrix Protein 1
DP	Dental Pulp
ECM	Extracellular Matrix
(M)DPSCs	(Mobilized) Dental Pulp Stem Cells
DSP	Dentin Sialoprotein
DSPP	Dentin Sialophosphoprotein
EDTA	Ethylenediaminetetraacetic Acid
ELISA	Enzyme-linked Immunosorbent Assay
EtOH	Ethanol
(b)FGF2	(basic) Fibroblast Growth Factors 2
G-CSF	Granulocyte Colony-Stimulating Factor
GF	Growth Factors
GFP	Green Fluorescent Protein
GIC	Glass Ionomer Cement
HE	Hematoxylin & Eosin
HG-MS	Heparin-conjugated gelatin nanospheres immobilized in poly-L-lactide microspheres
HUVEC	Human Umbilical Vein Endothelial Cell
HG	Hydrogel
HSP-25	Human Shock Protein-25
IF	Immunofluorescence
IGF	Insulin-like Growth Factor
IHC	Immunohistochemistry
IRM	Intermediate Restorative Material (product)
LC3	Microtubule-associated protein 1A/1B-Light Chain 3
MDP	Multi-Domain Peptide
MSC	Mesenchymal Stem Cell
MT	Masson’s Trichrome
MTA	Mineral Trioxide Aggregate
N.C.	Negative Control
NaOCl	Sodium hypochlorite
NGF	Nerve Growth Factor
PBMT	Photobiomodulation Therapy
PBS	Phosphate Buffer Saline
PCNA	Proliferating Cell Nuclear Antigen
PDGF	Platelet-Derived Growth Factor
PDL	Periodontal Ligament
PECAM	Platelet Endothelial Cells Adhesion Molecule
PLLA	Poly-L-Lactide
PICO	Population, Intervention, Comparison, Outcome
P.C.	Positive Control
PRISMA	Preferred Reporting Items for Systematic Reviews and Meta-Analyses
PRP	Protein-Rich Plasma
(q)PCR	(quantitative) Polymerase Chain Reaction
rCT	Regenerated Connective Tissue
REP	Regenerative Endodontic Procedures
rMT	Regenerated Mineralized Tissue
SAP	Self-Assembling Peptide
SCAP	Stem Cells from the Apical Papilla
SCF	Stem Cell Factor
SCID	Severe Combined Immunodeficient
SDF-1a	Stromal cell-Derived Factor 1a
SHED	Stem Cells from Human Exfoliated Deciduous Teeth
SLan	Self-Assembling Angiogenic Peptides
SLed	Self-Assembling Dentinogenic Peptides
SYRCLE	Systematic Review Centre for Laboratory Animal Experimentation
TAP	Triple Antibiotic Paste
TE	Tissue Engineering
TEM	Transmission Electron Microscope
TGF-b1	Transforming Growth Factor b1
TRH-DE	Thyrotropin-Releasing Hormone-Degrading Ectoenzyme
VEGF	Vascular Endothelial Growth Factor
vWF	von Willebrand Factor
(rh)Wnt3a	(Recombinant Human) Wingless Int-1 signaling protein 3a
